# Supplementary material for: Psilocybin desynchronizes the human brain
Source: Nature. 2024 Jul 17;632(8023):131–8. doi: 10.1038/s41586-024-07624-5 (PMC11291293; doi:10.1038/s41586-024-07624-5)
Supplement: Supplementary file 1 — This file contains Supplementary Table 1, Figs. 1–7 and Methods. [file 41586_2024_7624_MOESM1_ESM.docx]

# Supplementary Information

# Psilocybin desynchronizes the human brain

Joshua S. Siegel^1^, Subha Subramanian^2^, Demetrius Perry^1^, Benjamin P. Kay^3^, Evan M. Gordon^4^, Timothy O. Laumann^1^, T. Rick Reneau^4^, Nicholas V. Metcalf^3^, Ravi V. Chacko^5^, Caterina Gratton^6^, Christine Horan^7^, Samuel R. Krimmel^3^, Joshua S. Shimony^4^, Julie A. Schweiger^1^, Dean F. Wong^4^, David A. Bender^1^, Kristen M. Scheidter^3^, Forrest I. Whiting^3^, Jonah A. Padawer-Curry^8^, Russell T. Shinohara^9,10,11^ , Yong Chen^11^, Julia Moser^12,13^, Essa Yacoub^14^, Steven M. Nelson^12,15^, Luca Vizioli^14^,  Damien A. Fair^12,13,14,15^, Eric J. Lenze^1^, Robin Carhart-Harris^16,17^, Charles L. Raison^18,19^, Marcus E. Raichle^3,4,8,20,21^, Abraham Z. Snyder^3,4^, Ginger E. Nicol^1^* & Nico U.F. Dosenbach^3,4,8,20,22^*

*equal contribution

1: Department of Psychiatry, Washington University School of Medicine, St Louis, MO, USA.

2: Department of Psychiatry, Beth Israel Deaconess Medical Center, Boston, MA, USA.

3: Department of Neurology, Washington University School of Medicine, St Louis, MO, USA.

4: Mallinckrodt Institute of Radiology, Washington University School of Medicine, St Louis, MO, USA.

5: Department of Emergency Medicine, Advocate Christ Health Care, Oak Lawn, IL, USA.

6: Department of Psychology, Florida State University, Tallahassee, FL, USA.

7: Miami VA Medical Center, Miami, FL, USA.

8: Department of Biomedical Engineering, Washington University in St. Louis, St Louis, MO, USA.

9: Center for Biomedical Image Computing and Analytics, University of Pennsylvania, Philadelphia, PA, USA.

10: Penn Statistics in Imaging and Visualization Endeavor, Perelman School of Medicine, University of Pennsylvania, Philadelphia, PA, USA.

11: Department of Biostatistics, Epidemiology and Informatics, Perelman School of Medicine, University of Pennsylvania, Philadelphia, PA, USA.

12: Masonic Institute for the Developing Brain, University of Minnesota, Minneapolis, MN, USA.

13: Institute of Child Development, University of Minnesota, Minneapolis, MN, USA.

14: Center for Magnetic Resonance Research (CMRR), University of Minnesota, Minneapolis, MN, USA.

15: Department of Pediatrics, University of Minnesota, Minneapolis, MN, USA.

16: Department of Neurology, University of California, San Francisco, CA, USA.

17: Centre for Psychedelic Research, Imperial College London, London, UK.

18: Usona Institute, Fitchburg, WI, USA.

19: Department of Psychiatry, University of Wisconsin School of Medicine & Public Health, Madison, WI, USA.

20: Department of Psychological and Brain Sciences, Washington University in St. Louis, St Louis, MO, USA.

21: Department of Neuroscience, Washington University School of Medicine, St Louis, MO, USA.

22: Department of Pediatrics, Washington University School of Medicine, St Louis, MO, USA.

Corresponding Author:

Joshua S. Siegel

jssiegel@wustl.edu

Table of Contents

[Supplementary Tables and figures 3](#_Toc170734560)

[Supplementary Table 1. 3](#_Toc170734561)

[Supplementary Fig. 1. Network changes compared across different conditions, brain structures, and measures. 4](#_Toc170734562)

[Supplementary Fig. 2. Pulse and respiratory rates across conditions. 5](#_Toc170734563)

[Supplementary Fig. 3. Alternative methods for computing FC change. 6](#_Toc170734564)

[Supplementary Fig. 4. Comparison of analyses with and without PhysIO-based nuisance regression. 7](#_Toc170734565)

[Supplementary Fig. 5. Relationship of local FC change to score on each domain of the mystical experience questionnaire. 8](#_Toc170734566)

[Supplementary Fig. 6. Whole-brain FC changes for every scan. 9](#_Toc170734567)

[Supplementary Fig. 7. Effects of task on psilocybin-associated FC change and desynchronization after regressing out evoked responses. 10](#_Toc170734568)

[Supplementary Video Captions 11](#_Toc170734569)

[Supplementary Methods and Results 12](#_Toc170734570)

[Exclusion criteria 12](#_Toc170734571)

[Study screening 12](#_Toc170734572)

[Subjective and cognitive assessments 12](#_Toc170734573)

[Set and setting protocol 13](#_Toc170734574)

[Drug administration 13](#_Toc170734575)

[Treatment guess 13](#_Toc170734576)

[Concurrent medication use 13](#_Toc170734577)

[Data management 14](#_Toc170734578)

[Resting-state functional MRI processing, and surface projection 14](#_Toc170734579)

[Task fMRI analyses 15](#_Toc170734580)

[Physiological monitoring during fMRI 15](#_Toc170734581)

[Regression of evoked response in preprocessing 16](#_Toc170734582)

[Supplementary References 16](#_Toc170734583)

# Supplementary Tables and figures

## Supplementary Table 1.

Participant demographics and neuropsychological assessments

| Participant | P1 | P2 | P3 | P4 | P5 | P6 | P7 |
| --- | --- | --- | --- | --- | --- | --- | --- |
| Demographics | | | | | | | |
| Sex (self-report) | M | M | F | F | M | F | M |
| Age Range (years) | 41-45 | 36-40 | 36-40 | 36-40 | 18-20 | 21-25 | 41-45 |
| Weight (lbs) | 227 | 151 | 148 | 173 | 169 | 224 | 215 |
| Last degree completed | Bachelor | Bach. | Bach. | Graduate | High School | Bach. | Graduate |
| Last psychedelic exposure (months) | 24 | 24 | 12 | 24 | 24 | 12 | 60 |
| Replication protocol (days between doses) | Yes (273 days) | No | Yes (349 days) | Yes (350 days) | Yes (300 days) | No | No |
| Baseline Mini-International Personality Item Pool (Mini-IPIP) | | | | | | | |
| Neuroticism | 3 | 2.5 | 2.75 | 2 | 2 | 1.25 | 2.5 |
| Extraversion | 2 | 3.5 | 3.25 | 3.25 | 4 | 3 | 3.5 |
| Openness | 2.5 | 3.75 | 1.75 | 4.75 | 4 | 5 | 3.75 |
| Agreeableness | 3.75 | 4.25 | 2.5 | 4.5 | 4 | 4.5 | 4.25 |
| Conscientiousness | 3 | 1.75 | 3 | 4.25 | 3.75 | 2.5 | 1.75 |
| MRI data obtained | | | | | | | |
| MRI Visits (protocol + replication) | 19+5 | 21 | 18+3 | 15+4 | 14+4 | 12 | 14 |
| 15-minute rest fMRI scans, no drug | 53 | 44 | 40 | 44 | 41 | 26 | 28 |
| 15-minute rest fMRI scans, on PSIL | 6 | 2 | 5 | 5 | 7 | 2 | 6 |
| 15-minute rest fMRI scans, on MTP | 2 | 2 | 2 | 3 | 4 | 3 | 3 |
| Total task fMRI sessions | 9 | 9 | 7 | 9 | 3 | 6 | 16 |
| diffusion MRI scans, no drug | 12 | 12 | 12 | 16 | 16 | 16 | 8 |
| diffusion MRI scans, PSIL | 2 | 0 | 2 | 2 | 2 | 0 | 0 |
| diffusion MRI scans, MTP | 2 | 0 | 2 | 2 | 0 | 0 | 0 |
| Other protocol aspects | | | | | | | |
| Respirations and pulse acquired | Partial* | No | Partial* | Yes | Yes | Yes | Yes |
| Completed replication protocol | Yes | No | Yes | Yes | Yes | Yes | No |

**
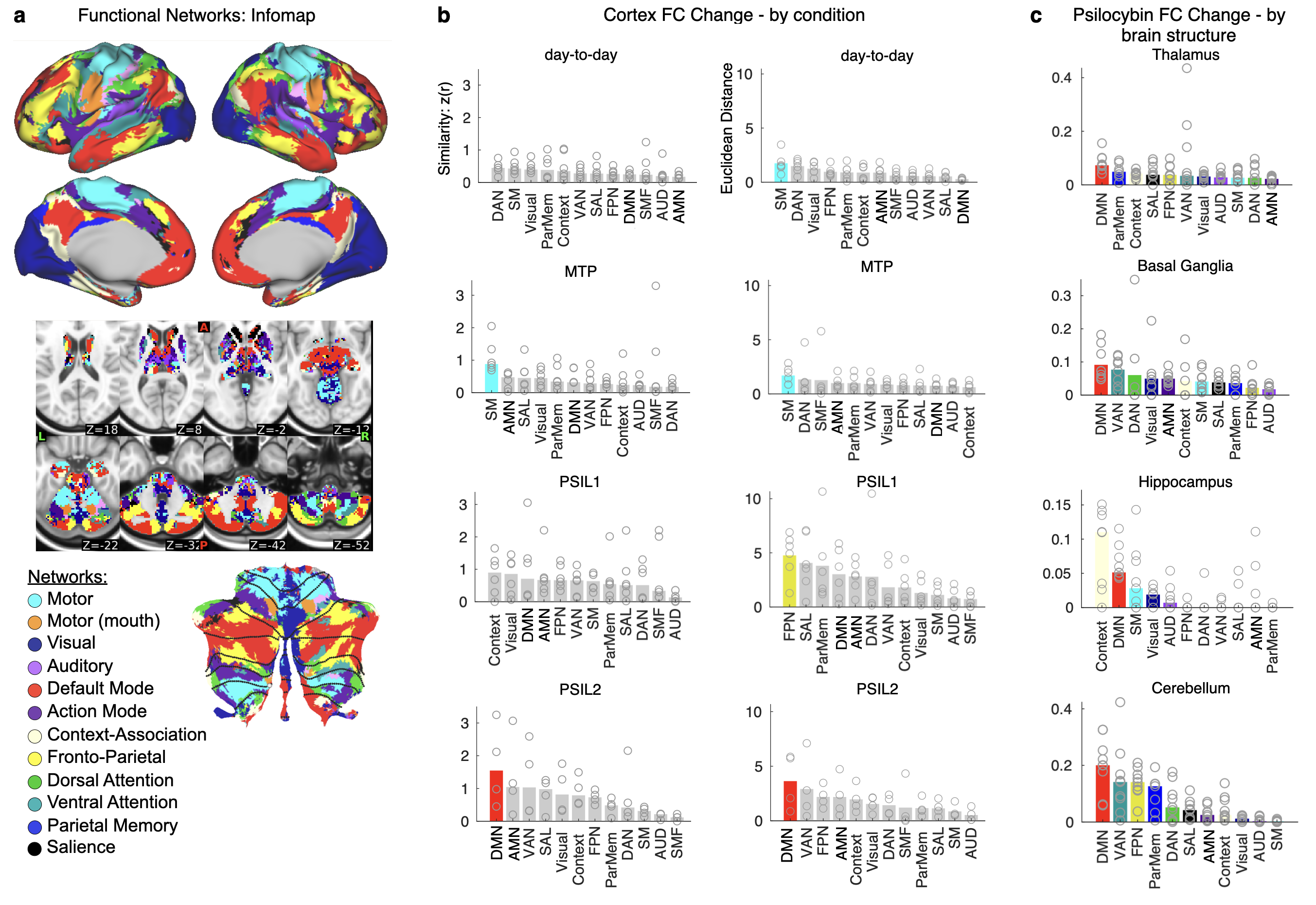
**

## Supplementary Fig. 1. Network changes compared across different conditions, brain structures, and measures.

**a)** Mode functional network map (*n* = 6 participants; Infomap-based). **b)** Network selectivity of cortical FC change is assessed for different conditions and separately for psilocybin (PSIL) initial and replication doses. Left column of bar plots shows FC change based on Euclidean distance, right column is based on (decrease in) bivariate correlation (similarity). Drug effects measured by Similarity (*z*(*r*)) values (right) are inverted to be consistent with distance (left). For day-to-day, methylphenidate (MTP) and PSIL, *n* = 6. For PSIL2 (replication dose) *n* = 4. Functional networks with FC change greater than expected by chance, based on permutation of network labels (spin test, 1,000 null rotation), are shown in their respective network colors (as in **a**). Similarity method: MTP *P*_SM_ = 0.001, PSIL2 *P*_DMN_ < 0.001, all other *P* > 0.05. Distance method: day-to-day *P*_SM_ = 0.048, MTP *P*_SM_ = 0.015, PSIL1 *P*_FPM_ < 0.001, PSIL2 *P*_DMN_ < 0.001, all other *P* > 0.05. **c)** Network selectivity of psilocybin-associated FC change (as shown in Fig. 1a) is assessed for different subcortical structures (here, network colors do not indicate any statistical test).


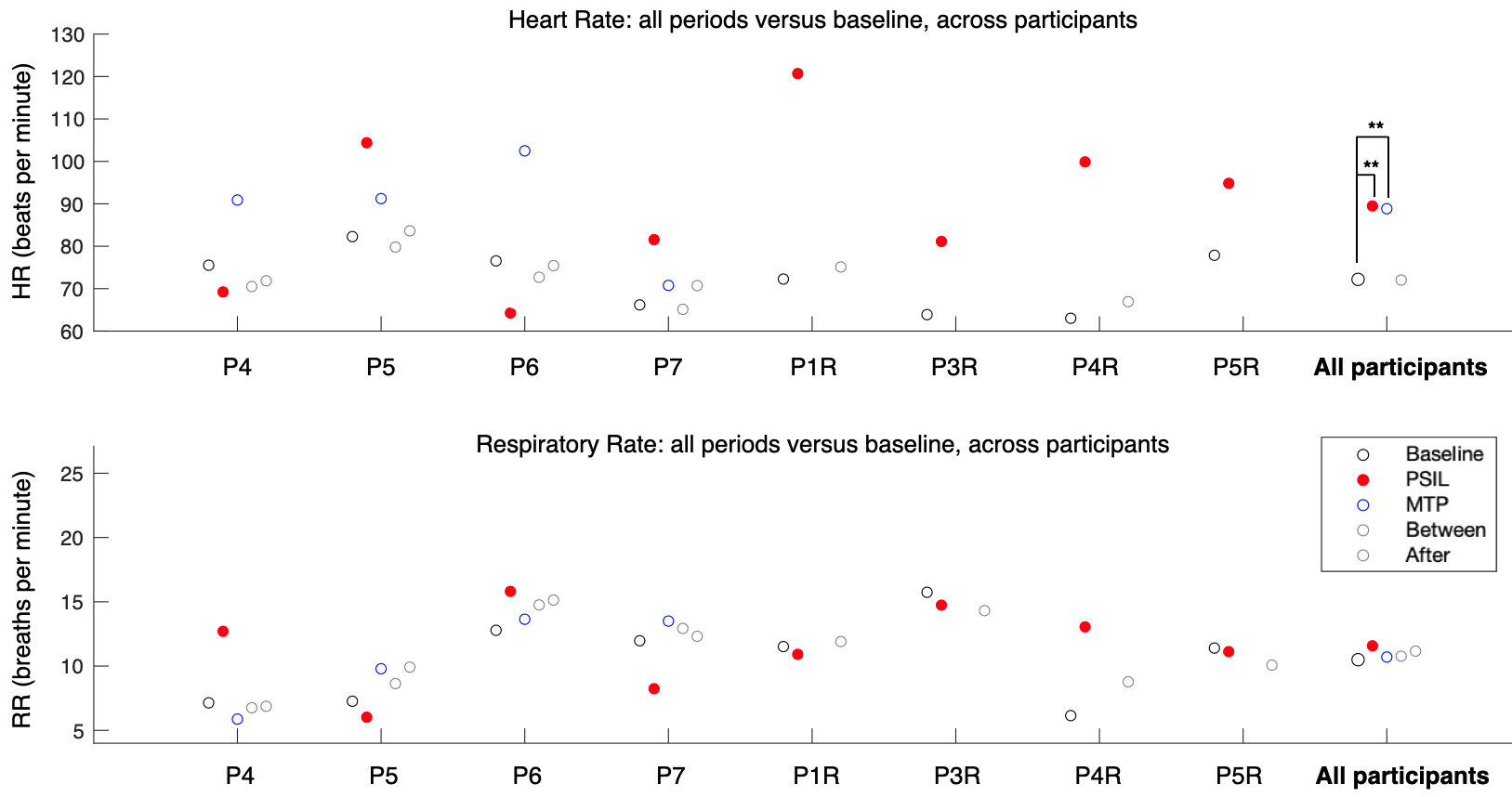


## Supplementary Fig. 2. Pulse and respiratory rates across conditions.

Mean heart rate (HR) and respiratory rate (RR) from continuous measurement during fMRI scans for each participant and all participants (average). A linear mixed effects model (see Methods) was used to test if a given condition differed significantly from baseline. HR = 199 observations, MTP-baseline *Estimate* (95% CI) = 16.7 bpm (11.0, 20.3), *t_(196)_* = 6.6, *P_uncorr_* = 3.12 x 10^-10^; PSIL-baseline *Estimate* (95% CI) = 21.1 bpm (16.6, 25.6), *t_(196)_* = 9.2, *P_uncorr_* = 4.04 x 10^-17^). No significant difference in HR was observed between MTP and PSIL (*P_uncorr_* = 0.399). ***P* < 0.001, uncorrected.


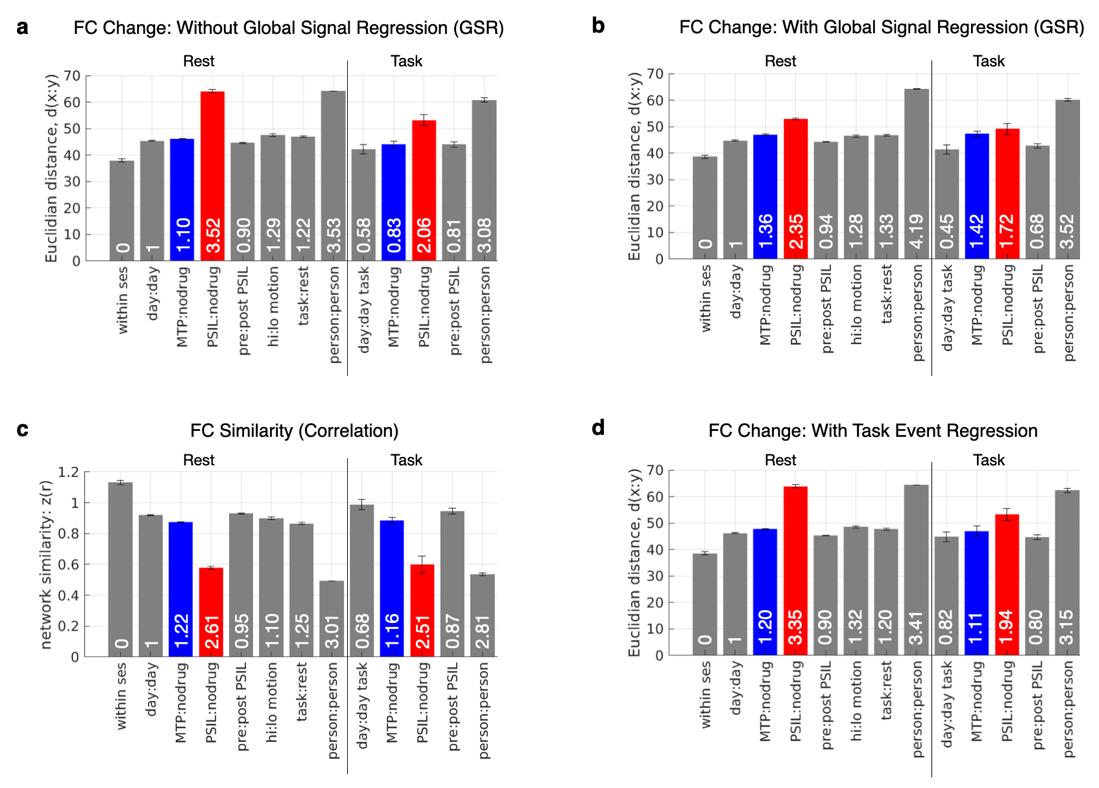


## Supplementary Fig. 3. Alternative methods for computing FC change.

FC change was defined as the average Euclidean distance between pairs of vectorized FC matrices, for a variety of conditions including: from the same individual within a single baseline session (70 pairs), from the same individual across baseline days (’day:day’, 672 pairs), from the same individual but during different drug states (MTP:nodrug 362 pairs, psil:no-drug 436 pairs), from the same individual but during different tasks (task:rest, 190 pairs), from the same individual comparing highest motion scans and baseline (hi:lo motion, 182 pairs), and from different individuals’ baseline (person:person, 3,635 pairs)***.*** Error bars indicate SEM. **a)** FC change, as reported in the manuscript. Individual datapoints for key comparisons are shown in Figure 1e**. b)** FC change after global signal regression (GSR). **c)** FC change calculated using ‘similarity’ (bivariate correlation) rather than difference, yielded similar results (note that change in similarity is inverted in panel b for comparison). **d)** FC change computed after the evoked responses were regressed out from task fMRI scans. The grounding effect of task performance remained (see Supplementary Figure 6).


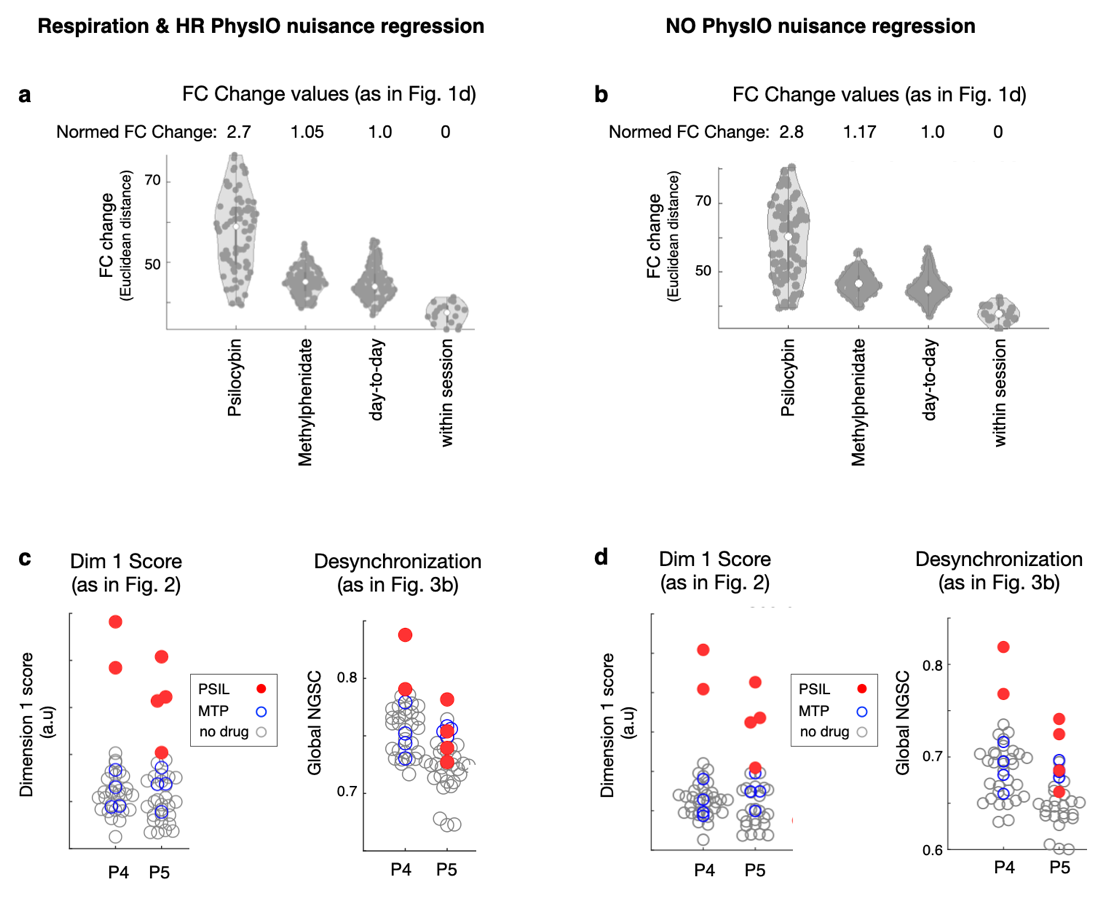


## Supplementary Fig. 4. Comparison of analyses with and without PhysIO-based nuisance regression.

Data shown are for a subset of participants with high quality pulse and respiratory traces. **a)** Whole-brain FC change scores, generated as in Fig. 1d by comparing every scan to baseline, but with PhysIO-based nuisance regression of respiratory rate (RR) and heart rate (HR). Normalized FC change values for each condition are listed above the violin plots. **b)** Same as in panel **a**, but without PhysIO-based nuisance regression, so exact same as in Fig. 1d, but for a subset of the date with usable physiological recordings data. **c)** Left, scores on Dim 1 - generated using multi-dimensional scaling on the entire cohort and then multiplying Dim1 weights by FC weights for each scan. (Dim1 score LME (linear mixed effects) model, PSIL (psilocybin) vs Baseline, *t_(93)_* = 4.5, *P_uncorr_* = 2.0 x 10^-5^). Right, desynchronization (Global NGSC (normalized global spatial complexity) LME, PSIL vs Baseline, *t_(93)_* = 3.0, *P_uncorr_* = 0.0034). All analyses with PhysIO-based nuisance regression. **d)** Same analyses as in panel **c**, but without PhysIO-based nuisance regression (Dim1 score LME, PSIL vs Baseline, *t_(93)_* = 4.1, *P_uncorr_* = 9.0 x 10^-5^; Global NGSC LME, PSIL vs Baseline, *t_(93)_* = 4.4, *P_uncorr_* = 3.1 x 10^-5^).

**
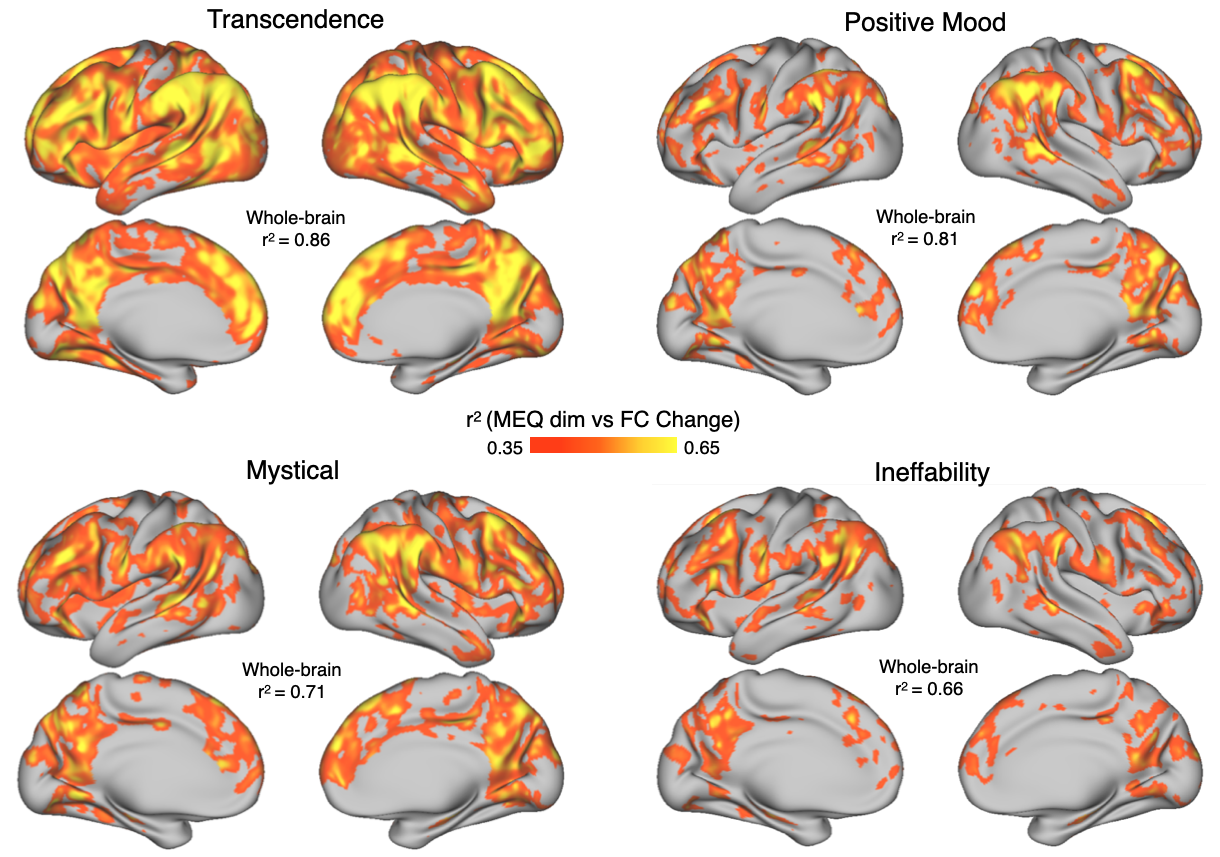
**

## Supplementary Fig. 5. Relationship of local FC change to score on each domain of the mystical experience questionnaire.

*r^2^* values are plotted across the cortical surface for average rating in each MEQ30 dimension across 16 drug doses (10 PSIL, 6 MTP). *r^2^* for whole-brain FC change is given in the middle. LME model: Transcendence *t_(13)_* = 5.6, *P_uncorr_* = 8.3 x 10^-4^; Positive mood *t_(13)_* = 3.7, *P_uncorr_* = 7.5 x 10^-3^; Mystical *t_(13)_* = 6.0, *P_uncorr_* = 8.3 x 10^-3^; Ineffability *t_(13)_* = 2.4, *P_uncorr_* = 0.048).


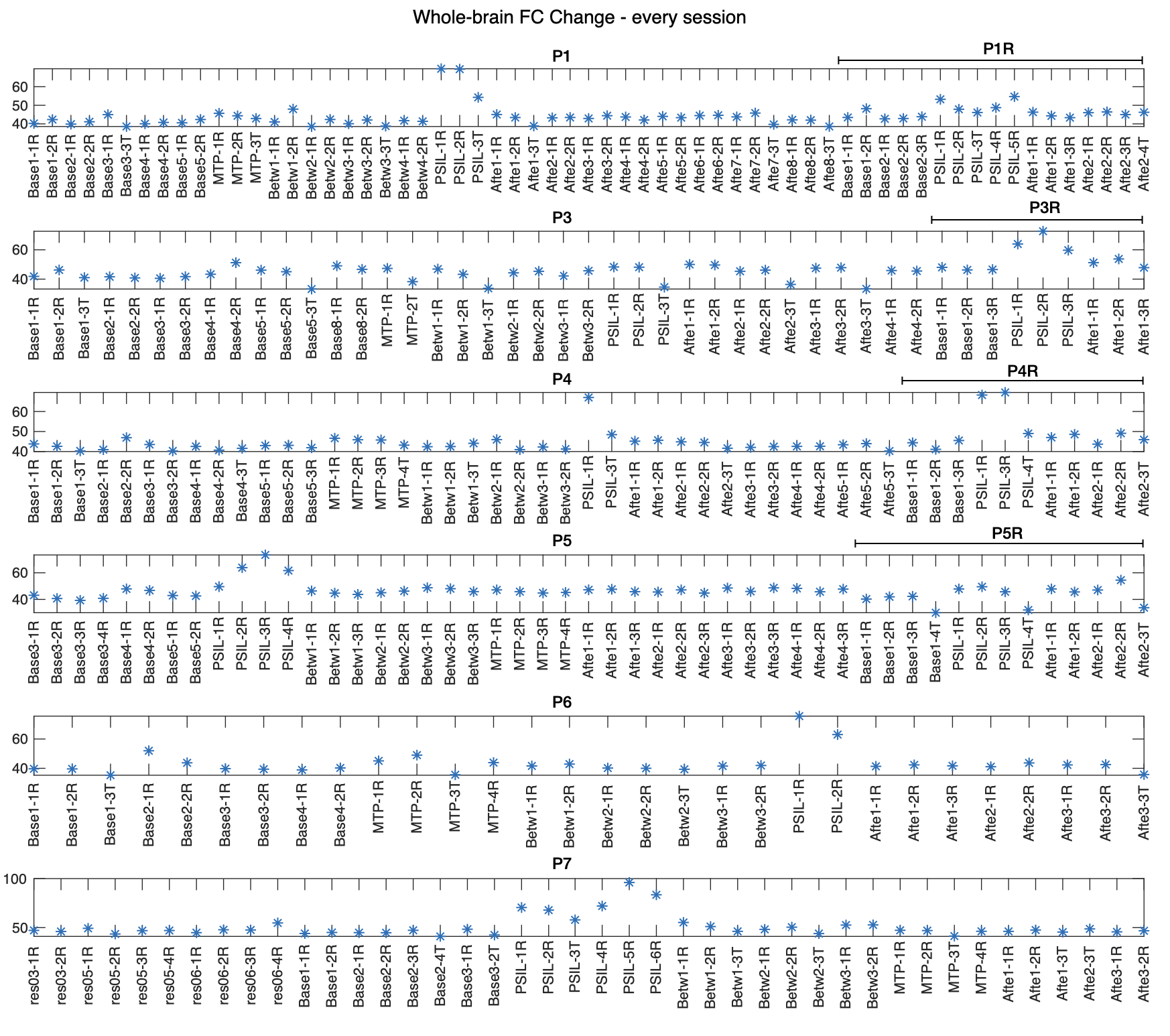


## Supplementary Fig. 6. Whole-brain FC changes for every scan.

Y-axes show FC change (Euclidean distance between vectorized FC matrices) between each scan and baseline. Labels underneath each scan indicate session, scan, and rest/task. For example, ‘Base3-2R’ indicates baseline visit 3, scan 2, rest; ‘MTP-3T’ indicates methylphenidate visit, scan 3, task.


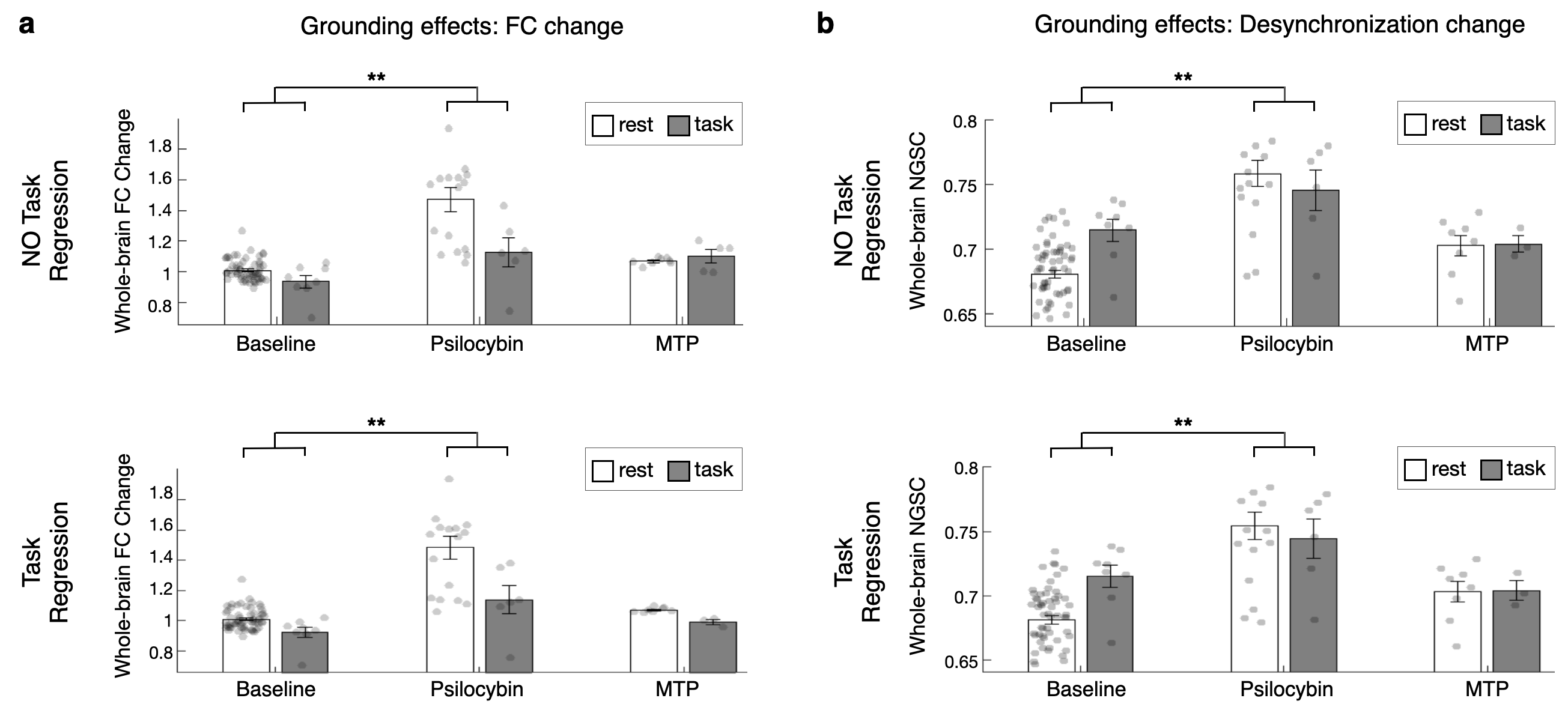


## Supplementary Fig. 7. Effects of task on psilocybin-associated FC change and desynchronization after regressing out evoked responses.

Paralleling Fig. 4, bar graphs indicate corresponding FC change and NGSC (normalized global spatial complexity) values from different rest/task and drug conditions. Linear mixed effects (LME) models testing an interaction of task x PSIL on FC Change in *n* = 7 with task data on PSIL. **a)** No Task Regression: *Estimate* (95% CI) = -6.48 (-9.59, -3.37), *t_(265)_* = -6.48, *P_uncorr_* = 5.49 x 10^-5^. *Estimate* (95% CI) = -6.48 (-9.59, -3.37), *t_(265)_* = -6.48, *P_uncorr_* = 5.49 x 10^-5^. Task regression: *Estimate* (95% CI) = -10.58 (-14.72, -6.43), *t_(265)_* = -5.02, *P_uncorr_* = 9.49 x 10^-7^. **b)** Interaction of task x PSIL on NGSC. No task regression: *Estimate* (95% CI) = -0.042 (-0.056, -0.027), *t_(265)_* = -5.62, *P_uncorr_* = 4.82 x 10^-8^. Task regression: *Estimate* (95% CI) = -0.0362 (-0.059, -0.013), *t_(265)_* = -3.08, *P_uncorr_* = 2.31 x 10^-3^. Bars indicate mean and error bars indicate SEM. ** *P* < 0.001, uncorrected.

## Supplementary Video Captions

**Supplementary Video 1|** Quality control plots for every fMRI scan. For each participant (P1, P3, P4, P5, P6, P7, concatenated) the quality control plots are concatenated in the order that the scans were acquired (see Extended Data Fig. 1). The top plot shows head position (frame-by-frame, relative to frame 1) separated into x-,y-,z-translation and x-,y-,z-rotation (6 parameters). The second plot from the top shows DVARS, which index the rate of change of fMRI signal across the entire brain at each frame of data. The D refers to temporal derivative of timecourses, and VARS refers to the root mean square variance over voxels. The third plot shows head motion measured as framewise displacement (FD) in mm. Underneath in the fourth row, the timecourse for the whole-brain grayordinates (cortex on top, subcortex on the bottom) are shown prior to pre-processing (known as ‘grayplot’ or ‘carpet plot’). The fifth row shows the same grayordinates, but after pre-processing (bandpass filtering, removal of nuisance signals by regression, and smoothing at 4 mm full width half max). The vertical black lines or bars in the grayplots indicate these data frames that were censored due to excessive head motion. At the end, quality control plots are compared to physiology (heart rate, respiratory rate) plots for every session in which physiological monitoring data were acquired.

**Supplementary Videos 2-7|** Timeseries of fully pre-processed resting-state fMRI (rs-fMRI) data (~9 minutes), taken from the first resting scan of the MRI session. Frame-by-frame rs-fMRI data, excluding high head motion frames (FD > 0.3 mm), are shown for the drug scans (psilocybin, methylphenidate) for each participant (P1, P3, P4, P5, P6, P7).

# Supplementary Methods and Results

## Exclusion criteria

Exclusion criteria included contraindications to MRI scanning (bone hardware, IUD, implantable devices) contraindications to psilocybin exposure (e.g., hypertension, cardiovascular disease, pregnancy); diagnosis of psychiatric condition (including substance use disorders); current use of certain psychotropic medication; previous adverse reactions to psychedelics (assessed with the Challenging Experience Questionnaire^1^; immediate family history of any schizophrenia spectrum disorder.

## Study screening

After prescreening and providing informed consent, participants underwent screening tests, including an electrocardiogram, urine drug screen, complete metabolic panel, and a urine pregnancy test. A study physician performed a physical exam and reviewed labs to ensure that participants did not have health conditions that would compromise their safety during the study. Once medically cleared, participants were scheduled for all planned imaging sessions and drug dosing days to ensure appropriate timing of pre-, post- and dosing day scans.

## Subjective and cognitive assessments

Assessments were conducted before, during, and after treatment sessions. This included subjective ratings, objective measures, personality survey and safety assessments. Assessments obtained are described below:

International Personality Item Pool-Five-Factor Model (Mini-IPIP): The Mini-IPIP is a 20-question survey administered to determine the Big Five factors of an individual’s personality: extraversion, agreeableness, conscientiousness, neuroticism, and openness to experience^2^.  The Mini-IPIP was administered at the following time points: baseline, post-drug one, post-drug two.

Mystical Experience Questionnaire (MEQ30): The MEQ30 is a 30-item self-report questionnaire that measures mystical experiences. It measures four factors: a) mystical (freedom from boundaries of one’s personal self and a feeling of unity to what is greater than one’s self), b) positive mood (sense of awesomeness or awe), c) transcendence of time and space (being outside of real of time), d) ineffability (sense that experience cannot be described well in words)^3,4^. The MEQ30 was administered at the following time points: baseline, post-drug one, post-drug two. We administered the MEQ30 on the same day of drug administration and the day after dosing day and determined that MEQ30 scores were similar on both days. Therefore, MEQ30 scores on dosing days were reported.

MEQ has shown an association to persistent effects and to symptom reduction across various conditions, including cancer-related distress, substance use disorder, and depressive disorders^5^. The 5D-ASC is considerably longer (96 vs 30 questions) and captures a wider variety of altered states of consciousness. Oceanic Boundlessness (OBN), one of five subdimensions of 5D-ASC, which includes subdimensions of experience of unity, spiritual experience, blissful state, and insightfulness, correlates strongly with MEQ^6^. OBN was shown to therapeutic efficacy of psilocybin in treatment-resistant depression^7^. We chose the MEQ30 because it has greater specificity to the effects of psychedelics and reduced participant burden.

Scores on the 4 domains of the MEQ30 (mystical, positive mood, transcendence of time and space, and ineffability) were computed by averaging ratings on questions within the domain. Correlation between domains were high (ranging from *r* = 0.81-0.94), consistent with prior report^3^.

## Set and setting protocol

Preparation and integration sessions were held in a dedicated research treatment room where the study drug was administered. Preparatory sessions were held one or two days before drug administration. Integration sessions were held one day after drug administration. The purpose of preparatory sessions was to build a therapeutic alliance between facilitators and participants. The participant’s personal history, developmental stage, current life situation, and intentions for and expectations of drug sessions were reviewed. Preparation and integration sessions occurred per Usona facilitator training guidelines.

## Drug administration

On dosing visits, following checking vitals, urine drug screen, and urine pregnancy test, participants received either 25 mg of psilocybin or 40 mg of methylphenidate. Both facilitators and participants were blinded. Medications were taken with lemon ginger tea. Following a 10-minute guided mindfulness meditation, participants were invited to lie on the sofa with eye shades as well as headphones and a curated music play list. One hour after drug administration, participants were transported to the MRI suite. Following the MRI, participants were transported back to the dedicated testing room and encouraged to direct their attention internally until subjective drug effects were resolved. When drug effects were resolved, study facilitators and participants completed post-dose questionnaires and a release checklist. Regardless of drug received, dosing sessions were 6-8 hours in length.

Heart rate and blood pressure were measured at regular intervals during dosing days (e.g., 30, 60, 90, 120, 240, 360, 420, and 480 minutes after drug ingestion). Subjects were also briefly queried about adverse effects during vital signs monitoring using an adverse events checklist. Rescue medications (risperidone for agitation, lorazepam for anxiety and niacin for chest pain) were available as needed. The Columbia Suicide Severity Rating Scale (C-SSRS) was used to assess for suicidal ideation and behavior during drug exposure^8^. Participants had access to a physician who was physically present throughout dosing day.

## Treatment guess

After each drug session (in the initial blinded cross-over portion of the study), participants were asked to guess if they had received psilocybin or methylphenidate. 6/7 participants correctly guessed which dose was psilocybin. Curiously, P3, the only participant who guessed incorrectly, showed a smaller FC change during psilocybin than any other participant (except for P5 replication dose, in which the participant vomited 30 minutes after swallowing the capsule).

## Concurrent medication use

Participants were asked to report any changes in prescription medications throughout the study protocol. None of the participants reported any changes in medication between initial and replication protocols. Four participants reported no concurrent medications, three participants reported at least one. P7 reported taking the same dose of Vyvanse throughout the study protocol.

## Data management

De-identified assessment scores, raw data from structural MRI and fMRI scans were uploaded into the Central Neuroimaging Data Archive (CNDA).

## Resting-state functional MRI processing, and surface projection

Preprocessing of fMRI data was done using an inhouse MRI processing pipeline and included: 1) removal of thermal noise using NORDIC (a local PCA approach in which temporal components of an fMRI signal that are indistinguishable from Gaussian noise are eliminated)^9^; 2) compensation for asynchronous slice acquisition using sinc interpolation; 3) compute linear spatial registration of all volumes within a run (using 4dfp tools); 4) elimination of odd/even slice intensity differences resulting from interleaved acquisition (debanding); 5) compute affine spatial registration across fMRI runs; 6) compute an run volume mean (of all low-noise volumes); 7) computation of field distortion on the basis of a spin echo field maps using FSL top-up^10^; and 8) gain field correction using FSL fast^11^ (computed on the run volume mean);

Resampling in MNI152 2 mm^3^ atlas space was accomplished for all echoes in one step combining (i) motion correction of volumes within a visit; (ii) distortion correction; (iii) gain field correction; (iv) affine spatial registration of average volumes across visits; and (v) non-linear MNI152 atlas registration via the fsl fnirt^12^. Optimal combination of echoes was then computed in MNI152 space using the weighted summation approach (as described in Posse et al113 equations 6 and 7), i.e.,

w (TEn) = $\frac{{TE}_{n}}{}$ exp(- $\frac{{TE}_{n}}{})$

where *TEn* is time of the n^th^ echo and time constant matches expected relaxation time $T_{2}^{*}$. Finally, the voxel-wise intensities were adjusted (one scalar per run) to obtain a mode value of 1,000 in the distribution of intensities summed over all voxels and volumes.

Following cross-modal registration, data were passed through several additional preprocessing steps: (i) tissue-based regressors were computed based on FreeSurfer segmentation^13^; (ii) temporal filtering to retain frequencies in the 0.009–0.08Hz band; and (iii) frame censoring (iv) removal by regression of the following signals that contain spurious variance: (a) six parameters obtained by rigid body correction of head motion (computed using inhouse 4dfp software), (b) signal from white matter, ventricles and extra-axial sources of noise (nuisance regressors were also bandpass filtered to match timecourse frequencies). Where indicated, respiratory and pulse-oximetry traces were used to generate additional physiological regressors using the PhysIO software package^14^.

The first four frames of each BOLD run were excluded. As has been reported previously, some participants exhibited high-frequency peaks in the power spectrum of head motion time courses, primarily in the phase-encoding (y) dimension. Thus, we low-pass filtered the y-translation estimate time courses at 0.1Hz in all participants prior to computing FD to prevent superfluous data loss^15–17^. We observed that this had minimal effect on the computation of FD (see FD computation with and without y-translation filtering in Supplementary Video 1). Frame censoring was implemented using framewise displacement^18^ with a threshold of 0.3 mm. This frame-censoring criterion was uniformly applied to all rs-fMRI data before functional connectivity computations. BOLD runs were excluded completely if they retained less than 50% usable frames after motion scrubbing.

Individualized cortical surfaces and subcortical volumes were generated for each participant’s T1 MRI using FreeSurfer automated segmentation. Segmentation errors were manually corrected. Following preprocessing, BOLD data were sampled to each participant’s individual cortical surface and subcortical volume using Connectome Workbench^19^.

Brain surface visualizations were generated using Connectome Workbench^19^.

## Task fMRI analyses

Task fMRI data were analyzed using a two-level approach. First solving a GLM for each session, second an ANOVA was used to test if evoked responses differed significantly between drug conditions (no drug, MTP, PSIL). fMRI data were preprocessed similar to resting data, with the exception of no nuisance regression and an FD threshold of 0.7 mm^20^. A generalized linear model was computed in two different ways: 1) vertexwise GLM, using an assumed hemodynamic response function to visualize the magnitude of task-evoked responses, 2) parcel-wise GLM, using a finite impulse response model to model evoked response for 13 TRs (22.89 seconds) after each trial. For (1), the canonical HRF from SPM (double gamma function) was used. For (2), a set of a priori regions of interest (ROIs) relevant to the task were selected from the Gordon-Laumann parcellation. These included: left/right calcarine sulcus (V1), left/right auditory cortex (A1), left language (Wernicke’s area), left hand knob, left angular gyrus, and right angular gyrus (default mode). Trial conditions (congruent, incongruent; button press, no button press) were collapsed to model a main effect of task. For both (1) and (2), additional regressors for button response, demean and detrend terms, and 6 movement parameters were added to generate a general linear model (GLM). This GLM was solved to estimate beta weights separately for each task visit.

In level 2 analyses, a two-way ANOVA was conducted using the ANOVAN function in MATLAB. This analysis allowed us to account for the effects of the drug (as a primary factor) as well as individual participants (as a secondary factor). A *P* value associated with the 'drug' factor of *P* < 0.05, would indicate that the drug has a significant effect on evoked response.

Of the 8 a priori regions on interest, only left and right V1 showed significant effect of drug (left V1 *P* = 0.03, right V1 *P* = 0.02, all other *P >* 0.1, uncorrected). Post-hoc comparison (Matlab: multcompare) for these regions indicated that left and right V1 showed differences in peak activation between non-drug and psilocybin conditions of *P* < 0.05.

## Physiological monitoring during fMRI

Recordings of pulse and respirations were added to the protocol prior to enrolling P4. Pulse and respiratory signals were recorded at 400 Hz with clocked timestamps. Physiological measurements were extracted using the PhysIO Toolbox^14^ as raw plethysmography signals. All signals were visually inspected and entire sessions were rejected if significant clipping occurred or if signals were noisy, without the regular oscillations expected in pulse or respiratory plethysmography. Instantaneous pulse rate (PR) and respiratory rate (RR) were determined by first labeling the peaks and troughs of the wave using Matlab’s findpeaks function, then calculating pulse rate or respiratory rate between each peak. This custom rate calculation agreed with PhysIO toolbox for respiratory rate but offered higher temporal resolution with respect to pulse rate. Physiological regressors for fMRI analyses were created using the automated PhysIO Toolbox.

Pulse and respiratory rates were analyzed across all participants and conditions (Supplementary Fig. 2). There was substantial variability in pulse rates across sessions. A mixed linear effects model was used to determine the relative effects of MTP and PSIL on the session means of physiological parameters. Because preliminary analyses suggested no change across no-drug conditions (baseline, between and after), all no-drug conditions were labeled the same in the LME. The average no-drug pulse rate was 72 beats per minute (95% CI: 68, 77 bpm). On average, MTP was associated with a 16.7 bpm increase (95% CI: 11.0, 20.3) in pulse rate (*P_uncorr_*= 3.12 x 10^-10^). PSIL was associated with a 21.1 bpm increase (95% CI: 16.6, 25.6) in pulse rate (*P_uncorr_* = 4.04 x 10^-17^). No significant difference in HR was observed between MTP and PSIL (*P_uncorr_* = 0.399).

The no-drug respiratory rate was 11 respirations per minute (95% CI: 9, 13 rpm) and there were no significant differences in respiratory rate across any conditions.

To assess if physiological confounds could explain observed psilocybin-associated FC changes, we selected the two participants with the highest quality pulse and respiratory data (P4, P5) and added physiological regressors during the nuisance regression step. This included 19 regressors generated from pulse-oximetry, respiratory belt, and their combination, generated using the PhysIO toolbox. The study results were not altered by the inclusion of PhysIO-generate regressors (Supplementary Fig. 4).

## Regression of evoked response in preprocessing

In our analyses of the effects of task on FC change and NGSC, and analyses of interactions between drug and task (Figure 4), evoked responses were not removed from timecourses prior to computing FC. To test if task evoked responses were affecting the observed results, we repeated the computation of whole-brain FC change and NGSC on timecourses following regression of evoked responses.

Specifically, SPM’s 3-parameter hemodynamic response function was convolved with the task design matrix plus a parameter coding response/non-response trials. These four timecourses were added to the list of regressors (along with other tissue- and movement-based nuisance regressors) prior to nuisance regression and smoothing. Whole-brain FC change and NGSC were then re-computed on residual timecourses for all task scans. We observed that the interaction of task with psilocybin was unaffected by regressing out evoked responses (Supplementary Fig. 7).

# Supplementary References

1. Barrett, F. S., Bradstreet, M. P., Leoutsakos, J.-M. S., Johnson, M. W. & Griffiths, R. R. The Challenging Experience Questionnaire: Characterization of challenging experiences with psilocybin mushrooms. *J. Psychopharmacol. (Oxf.)* **30**, 1279–1295 (2016).

2. Donnellan, M. B., Oswald, F. L., Baird, B. M. & Lucas, R. E. The mini-IPIP scales: tiny-yet-effective measures of the Big Five factors of personality. *Psychol. Assess.* **18**, 192–203 (2006).

3. Barrett, F. S., Johnson, M. W. & Griffiths, R. R. Validation of the revised Mystical Experience Questionnaire in experimental sessions with psilocybin. *J. Psychopharmacol. Oxf. Engl.* **29**, 1182–1190 (2015).

4. MacLean, K. A., Leoutsakos, J.-M. S., Johnson, M. W. & Griffiths, R. R. Factor Analysis of the Mystical Experience Questionnaire: A Study of Experiences Occasioned by the Hallucinogen Psilocybin. *J. Sci. Study Relig.* **51**, 721–737 (2012).

5. Ko, K., Knight, G., Rucker, J. J. & Cleare, A. J. Psychedelics, Mystical Experience, and Therapeutic Efficacy: A Systematic Review. *Front. Psychiatry* **13**, 917199 (2022).

6. Liechti, M. E., Dolder, P. C. & Schmid, Y. Alterations of consciousness and mystical-type experiences after acute LSD in humans. *Psychopharmacology (Berl.)* **234**, 1499–1510 (2017).

7. Roseman, L., Nutt, D. J. & Carhart-Harris, R. L. Quality of Acute Psychedelic Experience Predicts Therapeutic Efficacy of Psilocybin for Treatment-Resistant Depression. *Front. Pharmacol.* **8**, (2018).

8. Posner, K. *et al.* The Columbia–Suicide Severity Rating Scale: Initial Validity and Internal Consistency Findings From Three Multisite Studies With Adolescents and Adults. *Am. J. Psychiatry* **168**, 1266–1277 (2011).

9. Moeller, S. J. *et al.* Neural correlates of drug-biased choice in currently-using and abstinent individuals with cocaine use disorder. *Biol. Psychiatry Cogn. Neurosci. Neuroimaging* (2017) doi:10.1016/j.bpsc.2017.11.001.

10. Andersson, J. L. R., Skare, S. & Ashburner, J. How to correct susceptibility distortions in spin-echo echo-planar images: application to diffusion tensor imaging. *NeuroImage* **20**, 870–888 (2003).

11. Zhang, Y., Brady, M. & Smith, S. Segmentation of brain MR images through a hidden Markov random field model and the expectation-maximization algorithm. *IEEE Trans. Med. Imaging* **20**, 45–57 (2001).

12. Andersson, J. L., Jenkinson, M. & Smith, S. M. Non-linear registration aka Spatial normalization. (2007).

13. Fischl, B., Sereno, M. I., Tootell, R. B. H. & Dale, A. M. High-resolution intersubject averaging and a coordinate system for the cortical surface. *HUM BRAIN MAPP* **8**, 272–284 (1999).

14. Kasper, L. *et al.* The PhysIO Toolbox for Modeling Physiological Noise in fMRI Data. *J. Neurosci. Methods* **276**, 56–72 (2017).

15. Fair, D. A. *et al.* Correction of respiratory artifacts in MRI head motion estimates. *NeuroImage* **208**, 116400 (2020).

16. Gratton, C. *et al.* Removal of high frequency contamination from motion estimates in single-band fMRI saves data without biasing functional connectivity. *NeuroImage* **217**, 116866 (2020).

17. Raut, R. V., Mitra, A., Snyder, A. Z. & Raichle, M. E. On time delay estimation and sampling error in resting-state fMRI. *NeuroImage* (2019) doi:10.1016/j.neuroimage.2019.03.020.

18. Power, J. D. *et al.* Methods to detect, characterize, and remove motion artifact in resting state fMRI. *NeuroImage* **84**, 320–341 (2014).

19. Marcus, D. S. *et al.* Human Connectome Project informatics: Quality control, database services, and data visualization. *NeuroImage* **80**, 202–219 (2013).

20. Siegel, J. S. *et al.* Statistical improvements in functional magnetic resonance imaging analyses produced by censoring high-motion data points. *Hum. Brain Mapp.* **35**, 1981–1996 (2014).
