# Supplementary material for: Psilocybin desynchronizes the human brain
Source: Nature. 2024 Jul 17;632(8023):131–8. doi: 10.1038/s41586-024-07624-5 (PMC11291293; doi:10.1038/s41586-024-07624-5)
Supplement: Supplementary file 2 — Reporting Summary [file 41586_2024_7624_MOESM2_ESM.pdf]

nature portfolio

Corresponding author(s): Joshua Siegel

Last updated by author(s): May 2, 2024

Reporting Summary

Nature Portfolio wishes to improve the reproducibility of the work that we publish. This form provides structure for consistency and transparency in reporting. For further information on Nature Portfolio policies, see our Editorial Policies and the Editorial Policy Checklist.

Statistics

For all statistical analyses, confirm that the following items are present in the figure legend, table legend, main text, or Methods section.

n/a	Confirmed	
☐	☒	The exact sample size (*n*) for each experimental group/condition, given as a discrete number and unit of measurement
☐	☒	A statement on whether measurements were taken from distinct samples or whether the same sample was measured repeatedly
☐	☒	The statistical test(s) used AND whether they are one- or two-sided<br>*Only common tests should be described solely by name; describe more complex techniques in the Methods section.*
☐	☒	A description of all covariates tested
☐	☒	A description of any assumptions or corrections, such as tests of normality and adjustment for multiple comparisons
☐	☒	A full description of the statistical parameters including central tendency (e.g. means) or other basic estimates (e.g. regression coefficient) AND variation (e.g. standard deviation) or associated estimates of uncertainty (e.g. confidence intervals)
☐	☒	For null hypothesis testing, the test statistic (e.g. *F*, *t*, *r*) with confidence intervals, effect sizes, degrees of freedom and *P* value noted<br>*Give P values as exact values whenever suitable.*
☒	☐	For Bayesian analysis, information on the choice of priors and Markov chain Monte Carlo settings
☐	☒	For hierarchical and complex designs, identification of the appropriate level for tests and full reporting of outcomes
☐	☒	Estimates of effect sizes (e.g. Cohen's *d*, Pearson's *r*), indicating how they were calculated

Our web collection on statistics for biologists contains articles on many of the points above.

Software and code

Policy information about availability of computer code

Data collection	Task stimuli were presented using matlab psychtoolbox and custom code. Matching task fMRI code and stimuli are available at https://gitlab.com/siegelandthebrain1/Psilocybin_PFM/-/blob/main/image_task_clean.zip
Data analysis	Data processing code for the psilocybin PFM data (and Imperial College London LSD/psilocybin datasets) can be found here: http://128.252.217.203/repo/Pipeline/ (SVN Repository)<br>Code specific to psilocybin PFM task task analysis and manuscript analyses can be found here: https://gitlab.com/siegelandthebrain1/Psilocybin_PFM<br>Data processing code for the ABCD data can be found here: https://github.com/DCAN-Labs/abcd-hcp-pipeline<br>Software packages incorporated into the above pipelines for data analysis included:<br>Matlab R2019b, https://www.mathworks.com/ (including Psychtoolbox version 2.0 and Statistics and Machine Learning Toolbox version 11.6)<br>Cifti matlab utilities (including spin test): https://github.com/MidnightScanClub/SCAN<br>Connectome Workbench 1.5, http://www.humanconnectome.org/software/connectome-workbench.html<br>Freesurfer v6.2, https://surfer.nmr.mgh.harvard.edu/<br>FSL 6.0, https://fsl.fmrib.ox.ac.uk/fsl/fslwiki<br>4dfp tools, https://4dfp.readthedocs.io/en/latest/<br>Infomap, www.mapequation.org<br>CARET: http://brainvis.wustl.edu/

For manuscripts utilizing custom algorithms or software that are central to the research but not yet described in published literature, software must be made available to editors and reviewers. We strongly encourage code deposition in a community repository (e.g. GitHub). See the Nature Portfolio guidelines for submitting code & software for further information.

Data

Policy information about availability of data

All manuscripts must include a data availability statement. This statement should provide the following information, where applicable:

- Accession codes, unique identifiers, or web links for publicly available datasets
- A description of any restrictions on data availability
- For clinical datasets or third party data, please ensure that the statement adheres to our policy

All data from individual subjects P1-P7 is available upon completion of a data use agreement: https://wustl.app.box.com/folder/139716285911

The ABCD data used in this report came from ABCD the Annual Release 2.0, DOI 10.15154/1503209.

The Imperial College London psilocybin and LSD datasets (Carhart-Harris et al., 2012, Carhart-Harris et al., 2016) are available upon request.

Research involving human participants, their data, or biological material

Policy information about studies with human participants or human data. See also policy information about sex, gender (identity/presentation), and sexual orientation and race, ethnicity and racism.

Reporting on sex and gender	See supplemental Table 1 for P1-P7 characteristics<br>Male=4, Female=3<br>Imperial College LSD dataset: N=4/15 females (see Carhart-Harris et al, 2016)<br>Imperial College Psilocybin dataset: N=2/15 females (see Carhart-Harris et al, 2016)<br>ABCD: 49%M, 51%F
Reporting on race, ethnicity, or other socially relevant groupings	Not relevant/reported
Population characteristics	Details on age/education/past psychedelics use/personality are provided in Supplemental Table 1<br>Participant P1 P2 P3 P4 P5 P6 P7<br>Sex (self-report) M M F F M F M<br>Age Range (years) 41-45 36-40 36-40 36-40 18-20 21-25 41-45<br>Weight (lbs) 227 151 148 173 169 224 215<br><br>Carhart-Harris et al., 2012: n = 15 healthy adults (five females, mean age 34.1, SD 8.2)<br>Carhart-Harris et al., 2016: n = 20 participants completed the protocol but data were used for n = 15 (four females; mean age 30.5, SD 8.0)<br>ABCD: 9-10 year old males and females
Recruitment	WU P1-P7: Healthy adult participants were recruited from the Washington University community via word of mouth.<br><br>Imperial College Psilocybin/LSD: participants were recruited via word of mouth and provided written informed consent to participate.<br><br>ABCD: A very important motivation for the ABCD study is that its sample should reflect, as best as possible, the sociodemographic variation of the US population. The ABCD cohort recruitment emulates a multi-stage probability sample of eligible children: A nationally distributed set of 21 primary stage study sites, a probability sampling of schools within the defined catchment areas for each site, and recruitment of eligible children in each sample school. The major departure from traditional probability sampling of U.S. children originates in how participating neuroimaging sites were chosen for the study. Although the 21 ABCD study sites are well-distributed nationally the selection of collaborating sites is not a true probability sample of primary sampling units (PSUs) but was constrained by the grant review selection process and the requirement that selected locations have both the research expertise and the neuroimaging equipment needed for the study protocol.
Ethics oversight	P1-P7: All aspects of this study were approved by the Washington University School of Medicine Human Studies Committee and Institutional Review Board, the Food and Drug Administration (Center for Drug Evaluation and Research), and the Drug Enforcement Agency (which reviews all research protocol for the use of a Schedule 1 drug in humans).<br><br>Imperial College Psilocybin/LSD datasets: approved by the National Research Ethics Service committee London-West London and was conducted in accordance with the revised declaration of Helsinki (2000), the International Committee on Harmonization Good Clinical Practice guidelines, and National Health Service Research Governance Framework.<br><br>ABCD: The ABCD Study obtained centralized institutional review board approval from the University of California, San Diego, and each of the 21 study sites obtained local institutional review board approval.

Note that full information on the approval of the study protocol must also be provided in the manuscript.

Field-specific reporting

Please select the one below that is the best fit for your research. If you are not sure, read the appropriate sections before making your selection.

☒ Life sciences ☐ Behavioural & social sciences ☐ Ecological, evolutionary & environmental sciences

For a reference copy of the document with all sections, see nature.com/documents/nr-reporting-summary-flat.pdf

Life sciences study design

All studies must disclose on these points even when the disclosure is negative.

Sample size	Precision Functional Mapping focuses on within- rather than across-individual analysis, the following considerations were used to estimate sample size to measure persistent drug effects:<br><br>Conservative sample size calculation for single subject statistics: Based on priors from ketamine data, in a group of depression subjects imaged at two different baseline timepoints, the within-subject standard deviation of connectivity between the was 0.085. The average change in connectivity between sgACC and DMN two weeks after ketamine treatment was -0.04. This is an effect size of 0.47. The effect size for change in limbic FC was very similar, with within-subject SD = 0.072 and FC change after ketamine = -0.034. Given that this effect is measured on subjects was 12 minutes of data per imaging session, this is a lower limit of expected effect size. Based on this effect size (0.47), in order to have an 80% power of finding an effect with an error probability q <0.05 using a two-tailed t-test, we would need a total sample size of 38 fcMRI scan (19 before psilocybin, 19 after) in a single subject.<br><br>Optimistic sample size calculation for single subject statistics: The above power calculation is based on group-averaged data and standard regions of interest. By testing each hypothesis within-subject, we are able to substantially increase power by removing neurobiological variability (Gordon et al., 2017). It also gives the ability to more clearly identify individual variability in the effects of psilocybin. To our knowledge, only one published study has used this approach to examine the effects of temporary arm immobilization (casting) on brain plasticity (Newbold et al., 2020). They had N=3 subjects visit for 42-46 consecutive days and they acquired 30 minutes of resting fcMRI data per visit. They found, within 24 hours of casting, a very large effects of motor connectivity (left to right M1), with FC change of -0.23, -0.86, and -0.61, and in their three subjects (Fig. 3). Baseline within-subject motor FC variance was 0.0093, 0.0025, and 0.0058 for the same subjects. This yielded effect sizes of 2.4, 17.2, and 8.0, respectively. Assuming the smallest effect size of 2.4, we would require 5 MRI sessions per condition to have a 95% probability (power, 1-[illegible] err prob) to detect a significant effect.<br><br>While estimation of individual effects would be ideal, the primary endpoint will use a mixed-effects model in order to assess group effects. That statistical power of the primary endpoint should be approximately √N higher than the single subject t-test described above. Therefore, using the conservative effect size above, in order to have an 95% power of finding the hypothesized persisting effect of psilocybin at the group level, with an error probability a <0.05, we would need N=5 subjects.
Data exclusions	As stated in the methods, "One participant (P2) was not able tolerate fMRI while on psilocybin and had trouble staying awake on numerous fMRI visits after psilocybin and was excluded from analysis (except for data quality metrics in Extended Data Figure 1)." Otherwise, predetermined motion exclusion criteria were used to exclude motion-confounded fMRI data (see methods).<br><br>ABCD: 8,479 participants were selected as the participants with data available on stimulant use in the last 24 hours and at least 8 minutes of low-motion data, a pre-established criterion.
Replication	Experimental findings were replicated both within the original study cohort (4/7 participants returned 6-12 months after completion for a replication protocol) and across independently collected datasets from outside institutions (ABCD, Imperial College Psilocybin/LSD datasets)
Randomization	This was a randomized cross-over design. Participants underwent imaging during drug sessions with psilocybin (PSIL) 25mg, or methylphenidate (MTP) 40mg as well as non-drug imaging sessions. Randomization allocation was conducted via REDCap which displays randomization assignment to research team members who prepare study materials including drug or placebo but otherwise have no contact with participants.
Blinding	Participants and study staff (except for the study statistician running the randomization, and the study staff putting study medication into the capsule and bottle) were blinded to drug order. Active drug and placebo were in matching capsules.

Reporting for specific materials, systems and methods

We require information from authors about some types of materials, experimental systems and methods used in many studies. Here, indicate whether each material, system or method listed is relevant to your study. If you are not sure if a list item applies to your research, read the appropriate section before selecting a response.

Materials & experimental systems

n/a	Involved in the study	
☒	☐	Antibodies
☒	☐	Eukaryotic cell lines
☒	☐	Palaeontology and archaeology
☒	☐	Animals and other organisms
☐	☒	Clinical data
☒	☐	Dual use research of concern
☒	☐	Plants

Methods

n/a	Involved in the study	
☒	☐	ChIP-seq
☒	☐	Flow cytometry
☐	☒	MRI-based neuroimaging

Clinical data

Policy information about clinical studies

All manuscripts should comply with the ICMJE guidelines for publication of clinical research and a completed CONSORT checklist must be included with all submissions.

Clinical trial registration

NCT04501653

Study protocol

Abbreviated study protocol is avaiable at https://clinicaltrials.gov/study/NCT04501653. Full study IND protocol is available on request.

Data collection

Healthy young adults (N = 7, 18-45 years) were enrolled between April 2021 and March 2023 in a randomized cross-over precision functional brain mapping study at Washington University in Saint Louis.

Outcomes

Primary Objective (Predefined in IND protocol): Our overall goal is to use a Functional Connectivity (numerous visits, very long scans to produce individual connectomes) to examine the effects of psilocybin on cortical and cortico-subcortical brain networks that could explain its rapid and sustained behavioral effects.
1. Persisting changes Functional Connectivity [Time Frame: 2 weeks], measured with neuroimaging procedures outlined in the study methods.

Secondary Objective: Examine changes in blood flow, brain activity, and connectivity immediately following psilocybin. We will use methylphenidate and baseline fMRIs as control conditions.
1. Acute changes in functional connectivity and the hemodynamic response to neural activity [Time Frame: Immediate] will be measured with neuroimaging as outlined elsewhere in this protocol.
2. Participants will report a Mystical Experiences with psilocybin [Time Frame: 2-week], measured using Persisting Effects Questionnaire.

Plants

Seed stocks

Report on the source of all seed stocks or other plant material used. If applicable, state the seed stock centre and catalogue number. If plant specimens were collected from the field, describe the collection location, date and sampling procedures.

Novel plant genotypes

Describe the methods by which all novel plant genotypes were produced. This includes those generated by transgenic approaches, gene editing, chemical/radiation-based mutagenesis and hybridization. For transgenic lines, describe the transformation method, the number of independent lines analyzed and the generation upon which experiments were performed. For gene-edited lines, describe the editor used, the endogenous sequence targeted for editing, the targeting guide RNA sequence (if applicable) and how the editor was applied.

Authentication

Describe any authentication procedures for each seed stock used or novel genotype generated. Describe any experiments used to assess the effect of a mutation and, where applicable, how potential secondary effects (e.g. second site T-DNA insertions, mosiacism, off-target gene editing) were examined.

Magnetic resonance imaging

Experimental design

Design type

Stuctural sequences, resting state, and event-related task

Design specifications

Participants were scanned roughly every other day over the course of the experiment (Extended Data Fig. 1). Imaging was performed at a consistent time of day to minimize diurnal effects in functional connectivity. Neuroimaging was performed on a Siemens Prisma scanner (Siemens, Erlangen, Germany) in the neuroimaging labs (NIL) at the Washington University Medical Center.

Perceptual fMRI task: Participants also completed a previously validated event-related fMRI task. This was a suprathreshold auditory-visual matching task in which participants are presented with a naturalistic visual image (duration 500 ms) and coincident spoken English phrase and are asked to respond with a button press to indicate if the image and phrase are ‘congruent’ (for example, an image of a beach, and the spoken word beach) or ‘incongruent’.

Both accuracy and response time of button presses were recorded. Each trial was followed by a jittered inter-stimulus interval optimized for event-related designs. Two task fMRI scans were completed during a subset of imaging sessions. Task fMRI scans employed the same sequence used in resting fMRI, included 48 trials (24 congruent, 24 incongruent), and lasted a total of 410s. In analyses, the two task scans were concatenated to better match the length of the resting-state fMRI scans.

Behavioral performance measures

Performance was measure during the perceptual fMRI task.

Acquisition

Imaging type(s)

Structural (T1-w and T2w), Diffusion, Functional

Field strength

3.0T

Sequence & imaging parameters

MRI pulse sequences used to acquire the data are provided at https://gitlab.com/siegelandthebrain1/Psilocybin_PFM/-/blob/main/NP1161_MRI_sequence.pdf.
Structural scans (T1w and T2w) were acquired for each participant at 0.9 mm isotropic resolution, with real-time motion correction. Structural scans from different sessions were averaged together for the purposes of Freesurfer segmentation and nonlinear atlas registrations.
For functional scans, to capture high resolution images of blood oxygenation level-dependent (BOLD) signal, we used an echo-planar imaging sequence with 2mm isotropic voxels, multi-band 6, multi-echo 5 (TEs: 14.20 ms, 38.93 ms, 63.66 ms, 88.39 ms, 113.12 ms)90, TR 1761ms, flip angle = 68 degrees, and in-plane acceleration91 (IPAT/grappa) = 2. This sequence acquired 72 axial slices (144mm coverage). Each resting scan included 510 frames (lasting 15:49 minutes) as well as 3 frames at the end used to provide estimate electronic noise.
Every session included at least two 15-minute resting-state fMRI scans during which participants were instructed to hold still and look at a white fixation crosshair presented on a black background. Head motion was tracked in real time using Framewise Integrated Real-time MRI Monitoring software (FIRMM)92 . An eye-tracking camera (EyeLink, Ottawa) was used to monitor participants for drowsiness.

Area of acquisition

Whole-brain

Diffusion MRI ☐ Used ☒ Not used

Preprocessing

Preprocessing software

FSL 6.0 software tools used: FAST, Eddy, Topup, DTIFit, FEAT
Freesurfer versions 5.0, 5.3, and 6.0, recon-all pipelines for brain segmentation
Connectome workbench v1.0 and 1.5
4dfp tools (https://4dfp.readthedocs.io/)
Processing pipelines used:
https://github.com/DCAN-Labs/abcd-hcp-pipelines
https://github.com/DCAN-Labs/nhp-abcd-bids-pipeline
Smoothing kernels employed: from 2mm to 6mm FWHM in humans (geodesic on cortical surface)

Normalization

WU P1-P7: BOLD->T2 rigid body linear, T2->T1 rigid body linear, T1-> atlas nonlinear

Normalization template

MNI152

Noise and artifact removal

The acquisition and preprocessing of data were optimised for noise and artifact removal. This included:
- Preprocessing of fMRI data included: 1) removal of thermal noise using NORDIC (a local PCA approach in which temporal components of an fMRI signal that are indistinguishable from Gaussian noise are eliminated)111; 2) compensation for asynchronous slice acquisition using sinc interpolation; 3) compute affine spatial registration of all volumes within a run; 4) elimination of odd/even slice intensity differences resulting from interleaved acquisition (debanding); 5) compute affine spatial registration across fMRI runs; 6) compute an run volume mean (of all low-noise volumes); 7) computation of field distortion on the basis of a spin echo field maps using FSL top-up131; and 8) gain field correction using FSL fast132 (computed on the run volume mean);
- Resampling in MNI152 2 mm3 atlas space was accomplished for all echoes in one step combining (i) motion correction; (ii) distortion correction; (iii) gain field correction; (iv) linear registration of average volumes across visits; and (v) non-linear MNI152 atlas registration via the fsl fnirt133. Optimal combination of echoes was then computed in MNI152 space using the weighted summation approach (as described in Posse et al114 equations 6 and 7). Finally, the voxel-wise intensities were adjusted (one scalar per run) to obtain a mode value of 1000 in the distribution of intensities summed over all voxels and volumes.
- Following cross-modal registration, data were passed through several additional preprocessing steps: (i) tissue-based regressors were computed based on FreeSurfer segmentation134; (ii) temporal filtering to retain frequencies in the 0.009–0.08Hz band; and (iii) frame censoring (iv) removal by regression of the following signals that contain spurious variance: (a) six parameters obtained by rigid body correction of head motion, (b) signal from white matter, ventricles and extra-axial sources of noise (nuisance regressors were also bandpass filtered to match timecourse frequencies). Where indicated, respiratory and pulse-oximetry traces were used to generate additional physiological regressors using the PhysIO software package135.
- Individualized cortical surfaces and subcortical volumes were generated for each participant’s T1 MRI using FreeSurfer automated segmentation . Segmentation errors were manually corrected. Following preprocessing, BOLD data were sampled to each participant’s individual cortical surface and subcortical volume using Connectome Workbench140.
- In an alternative analysis, multi-echo ICA (ME-ICA) denoising designed to isolate spatially structured T2*- (neurobiological; “BOLD-like”) and S0-dependent (non-neurobiological; “not BOLD-like”) signals was performed using a modified version of the

"tedana.py" workflow (https://tedana.readthedocs.io/en/latest/). We found that this tool did not produce substantially different results from our processing pipeline.

Volume censoring

High motion time-points were censored using a frame-wise displacement (FD) threshold of 0.3mm.

Statistical modeling & inference

Model type and settings

Task fMRI was analyzed using a two-level approach. First solving a GLM for each session, second an ANOVA was used to test if evoked responses differed significantly between drug conditions (no drug, MTP, PSIL). fMRI data were preprocessed similar to resting data, with the exception of no nuisance regression and an FD threshold of 0.7 mm111. A generalized linear model was computed in two different ways: 1) vertexwise GLM, using a assumed hemodynamic response function to visualize the magnitude of task-evoked responses. 2) parcel-wise GLM, using a finite impulse response model to model evoked response for 19.37s after each trial. A set of a priori regions of interest (ROIs) relevant to the task were selected from the Gordon-Laumann parcellation. These included: left/right calcarine sulcus (V1), left/right auditory cortex (A1), left language (Wernicke's area), left hand knob, left angular gyrus, and right angular gyrus (default mode). Trial conditions (congruent, incongruent; button press, no button press) were collapsed to model a main effect of task. Additional demean and detrend terms and 6 movement parameters111 were added to the generate a general linear model (GLM). This GLM was solved to estimate beta weights separately for each task visit.
In level 2 analysis, a two-way ANOVA was conducted using the anovan function in MATLAB. This analysis allowed us to account for the effects of the drug (as a primary factor) as well as individual subjects (as a secondary factor). A p-value associated with the 'drug' factor of P<0.05, would indicate that the drug has a significant effect on evoked response.

Effect(s) tested

Main effects of psilocybin, effect of methylphenidate, and effect of pre- vs post-psilocybin, and interaction of task*psilocybin were tested on FC change, global normalized global spatial complexity, local global normalized global spatial complexity, anterior hippocampal FC change, heart rate, and respiratory rate were tested using a linear mixed effects model.

Linear mixed effects model takes advance of the nested levels in our multi-level precision functional mapping study design. Every scan was labeled on the following dimensions: Subject ID, MRI visit, task (task/rest), drug condition (pre-psilocybin, PSIL, MTP, post-psilocybin), and head motion (average framewise displacement, FD). rs-fMRI metrics (described below) were set as the dependent variable, drug (drug condition), task, FD (motion), and drug*task were defined as fixed effects, and Subject ID and MRI session were random effects.

Subjective experience was assessed for drug sessions using the mystical experience questionnaire (MEQ; see Supplementary methods). We applied a LME model across all drug sessions, similar to the one described above, but with MEQ total score as the dependent variable. Whole-brain FC change and framewise displacement (FD) were modeled as fixed effects, and participant was modeled as a random effect. The same model was solved using FC change from every vertex to generate a vertexwise map of the FC change versus MEQ.

Specify type of analysis: ☐ Whole brain ☐ ROI-based ☒ Both

Anatomical location(s)

MNI152 atlas was used to determined anatomical locations (e.g, MNI coordinates: anterior hippocampus loci -24, -22, -16 and 24,-18, -16

Statistic type for inference

(See Eklund et al. 2016)

FC change ('distance') was calculated at the vertex level to generate FC change maps and a linear mixed effects model (Eq. 1) was employed in combination with wild bootstrapping100,101 and threshold-free cluster enhancement (TFCE)86,102 to estimate P values for t-statistic maps resulting from the model (Fig. 1a-d, Fig. 4).

First, a FC change map was generated for every scan by computing, for each vertex, the average distance between its FC seedmap and the FC seedmap for each of that subject's baseline scans. Since each participant had multiple baseline visits, FC change was computed for baseline scans by computing distance from all other baseline scans (excluding scans within the same visit). This provided a measure of day-to-day variability. Second, the distance value was used as the dependent variable yij in the LME model to generate a t-statistic. Third, a wild bootstrapping procedure was implemented as follows. A large number of bootstrap samples (B = 1,000) were generated using the Rademacher procedure101, where the residuals were randomly inverted. Specifically, a Rademacher vector was generated by randomly assigning -1 or 1 values with equal probability to each observation's residual. By element-wise multiplication of the original residuals with the Rademacher vector, bootstrap samples were created to capture the variability in the data.

For the observed t-statistic-map, and each bootstrap sample, the TFCE algorithm was applied to enhance the sensitivity to clusters of significant voxels or regions while controlling for multiple comparisons. The value of the enhanced cluster statistic, derived from the bootstrap samples, was used to create a null distribution under the null hypothesis. By comparing the original observed cluster statistic with the null distribution, P values were derived to quantify the statistical significance of the observed effect. The P values were obtained based on the proportion of bootstrap samples that produced a maximum cluster statistic exceeding the observed cluster statistic.

The combined approach of wild bootstrapping with the Rademacher procedure and TFCE provided method to estimate p-values for our multi-level (drug condition, subject, session, task) design. This methodology accounted for the complex correlation structure, effectively controlled for multiple comparisons, and accommodated potential autocorrelation in the residuals through the Rademacher procedure. By incorporating these techniques, association with psilocybin and other conditions were reliably identified amidst noise and spatial dependencies.

Task fMRI: ROI-wise t-values converted to Z-scores were used for inference on task evoked response.

Correction

see above

Models & analysis

n/a	Involved in the study
☐	☒ Functional and/or effective connectivity
☒	☐ Graph analysis
☒	☐ Multivariate modeling or predictive analysis

Functional and/or effective connectivity

For P1-P7 particpants, a vertex/voxelwise functional connectivity matrix was calculated from the resting-state fMRI data for each 15-minute scan as the Fisher-transformed pairwise correlation of the timeseries of all vertices/voxels in the brain.

For whole brain and region-of-interest (ROI) based analysis, we used 385 surface/subcortical regions of interest. For cortical regions, we used a parcellation and community assignments generated by Gordon & Laumann and colleagues. For subcortical regions, we used a set of regions of interest generated to achieves full coverage and optimal region homogeneity. A subcortical limbic network was defined based on neuroanatomy: amygdala, antero-medial thalamus, nucleus accumbens, anterior hippocampus, posterior hippocampu. These regions were expanded to cover anatomical structures (e.g. anterior hippocampus).
To generate region-wise connectivity matrices, time courses of all surface vertices or subcortical voxels within a region were averaged. Functional connectivity (FC) was then computed between each region timeseries using Fisher z-transformed bivariate correlation.

The same ROI based approach was used on Imperial College London LSD/Psilocybin.

In the ABCD, parcel-wise group-averaged functional connectivity matrices were constructed for each participant by first calculating the parcel-wise functional connectivity within each participant as the Fisher-transformed pairwise correlation of the timeseries of all 385 regions on interest in the brain.
